# Into the Holocene, anatomy of the Younger Dryas cold reversal and preboreal oscillation

**DOI:** 10.1038/s41598-024-53591-2

**Published:** 2024-02-07

**Authors:** Jesse Velay-Vitow, Deepak Chandan, W. Richard Peltier

**Affiliations:** Department of Physics, University of Toronto, Toronto, ON Canada

**Keywords:** Palaeoclimate, Climate and Earth system modelling

## Abstract

During the most recent deglaciation, the upwards trend of warmer Northern Hemisphere (NH) temperatures was punctuated by a rapid and intense return to glacial conditions: the Younger Dryas (YD). The end of this event marks the beginning of the Holocene. Using the University of Toronto version of CCSM4, a model of the climate prior to the YD was created with correct boundary conditions. Various amounts of freshwater forcing were then applied to the Beaufort Gyre for forcing intervals ranging from 1 to 125 years. In several cases, this was sufficient to collapse the Atlantic Meridional Overturning Circulation (AMOC) and cause significant cooling over the NH. Crucially, after the forcing was ceased, the AMOC stayed in an off state for approximately a millennium before mounting a rapid recover to pre-YD levels. This recovery, which permanently reduced the extent of NH sea ice, occurred through the mechanism of a Polynya opening in the Irminger Sea during winter and led to a pronounced “overshoot” of the AMOC, during which NH temperatures were higher than before the YD.

## Introduction

The Younger Dryas (YD; $\sim$ 12.9 to 11.7 kya)^1^ was a rapid return to cold glacial conditions that occurred during the most recent deglaciation of the late Quaternary and marks the beginning of the Holocene. It has been the subject of intense inquiry for over 100 years since evidence of its existence was first discovered in the Allerød clay pits^2^. Escape from the YD was equally rapid and was conterminous with Meltwater Pulse 1b (MWP1B), which was a rapid rise in the eustatic sea level^3–5^. Other notable climatic events such as the preboreal oscillation, which was a series of climate fluctuations that began at approximately 11.3 ka^6,7^, and the onset of the early Holocene African Humid Period (AHP)^8^, are also related to the termination of the YD and have left an indelible mark on the climatic and anthropological evolution of the early Holocene.

There has been significant debate on the origin of the YD (e.g. see Broecker 1960^9^). A consensus has emerged that a release of glacial meltwater^10,11^ formed a freshwater cap over the North Atlantic^12^ which caused an AMOC shut down^13,14^ nearly as strong as during Heinrich Stadial 1^15^. There was initial debate over the route^16,17^, but the Mackenzie River is now favoured^18–22^. See Supplementary Materials for a full discussion of the history of the YD. For over two decades, attempts have been made to simulate the YD using global climate models (GCMs). Though the complexity of the GCMs employed has increased over this period, all explanations have involved the application of freshwater forcing to mimic the outburst flood now known to have caused the YD^17^. Manabe and Stouffer produced a series of papers using a global climate model to represent the YD. Their first attempt^23^ used a 4.5$^{\circ }$ resolution coupled atmosphere–ocean model in which a 10-year freshwater pulse of 1 Sv strength was applied to the high latitude North Atlantic, that caused a reduction in the Thermohaline Circulation (THC) and NH temperatures. However, the THC and temperatures recovered quickly when the forcing was arrested. Their second attempt^24^ involved a much longer pulse of 500 years at the lower rate of 0.1 Sv, which was intended to produce the 1000-year duration of the YD by causing a shutdown of the AMOC. Finally, Manabe and Stouffer included a heat flux land model in their GCM^25^, and repeated their earlier experiments. AMOC sensitivity experiments have employed the same approach of applying freshwater forcing to various locations to investigate the effect on AMOC strength. In Dahl et al.^26^, which employed the GFDL R30 coupled ocean-atmosphere GCM, a 100-year 0.1 Sv pulse was found to result in a 40% reduction in AMOC strength when applied to the North Atlantic. It was shown^27,28^ using the NCAR CCSM3 model, that freshwater forcing applied over the Arctic Ocean would be as effective in diminishing the strength of the AMOC as would the application of freshwater forcing directly over the regions of deep water formation. In particular, it has yet to be shown that a modern coupled atmosphere–ocean–sea ice model is able to accurately reproduce the climate changes that accompanied the YD-PB phenomenon.

Manabe and Stouffer^23–25^ arguably provided the most important insight into the cause of the climate impacts associated with YD by demonstrating that the application of freshwater forcing over the North Atlantic region where deep water forms would lead to a dramatic reduction of NH air temperatures. Their important work was nevertheless flawed in several ways that are corrected in the work described in what follows. Here, we demonstrate the important role played by the incorporation of an accurate model of continental ice cover and the influence of its high albedo, as well as a detailed model of sea ice dynamics, accurate atmospheric trace gases, and top-of-the-atmosphere radiative forcing. In “Methods” section, we provide a detailed discussion of these components of the climate model upon which our analyses of YD climate evolution are based. Given these significant improvements in the climate model we employ, our focus in this work is upon the strength and duration of the freshwater forcing that was responsible for the YD cold reversal when this is applied over the Beaufort Gyre of the Arctic Ocean as required by proxy evidence^17^.

**Figure 1:**
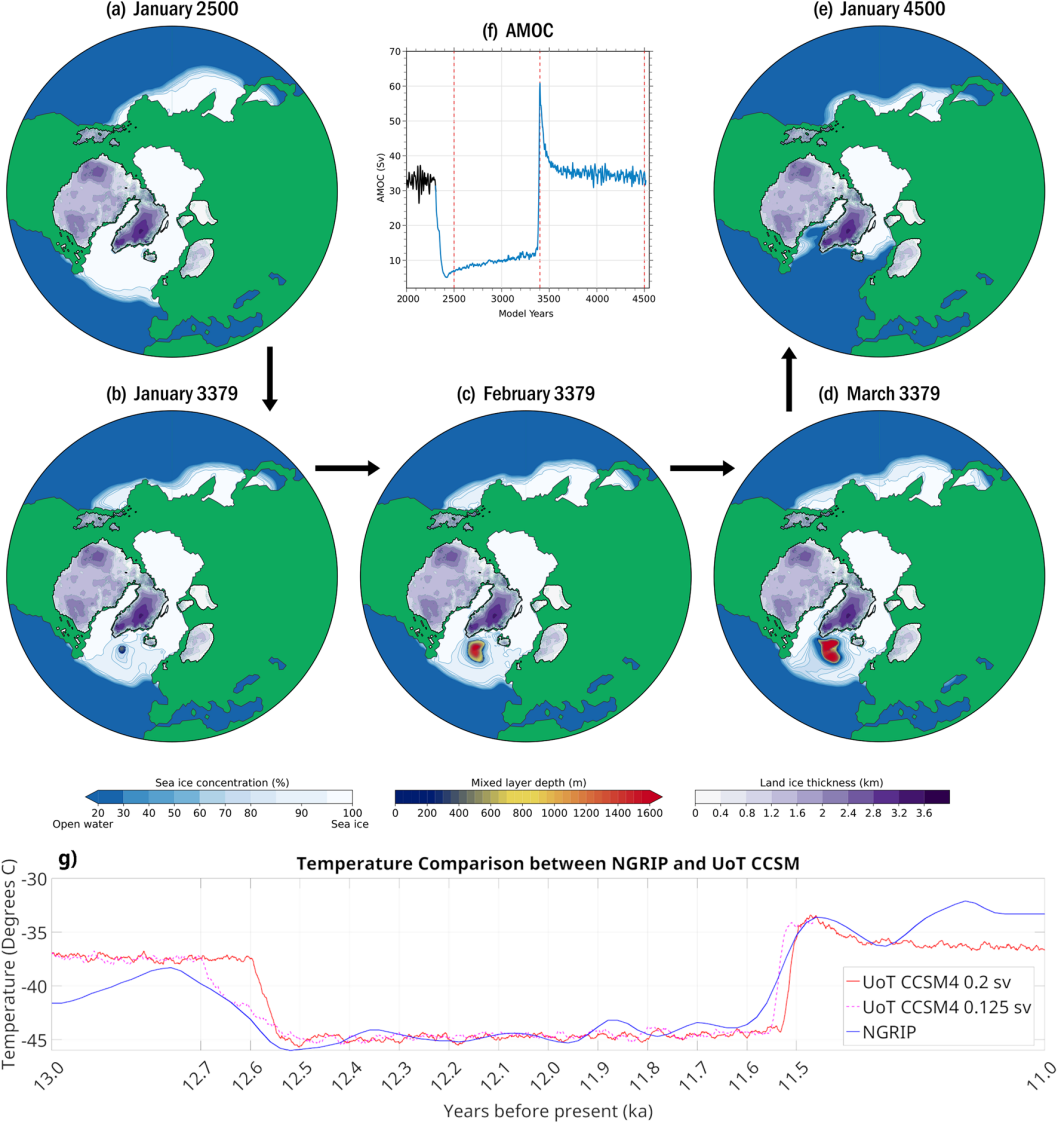
The rapid onset of and recovery from the YD is depicted by the time series of AMOC strength. The recovery occurs in precisely the same manner as in the recovery from cold stadial to warm interstadial conditions in a Dansgaard–Oeschger oscillation. Shown here in panel (**a**), the sea ice is at its maximum extent after the AMOC has collapsed following the injection of fresh water over the Beaufort Gyre. In panels (**b**–**d**) a polynya is seen to open rapidly in the Irminger Sea Basin. Finally, in panel (**e**), the sea ice extent is seen to be dramatically reduced after AMOC recovery. Terrestrial ice thickness is shown alongside sea ice percentage and, where applicable, mixed layer depth. Panel (**f**) shows the strength of the AMOC with dashed red lines indicating the times of the previously described panels. In panel (**g**) the comparison of model surface temperature and surface temperature inferred from the NGRIP ice core is presented. We show both the HF0.2_100 (solid red) and HF0.125_115 (dashed magenta) to illustrate that under different conditions our model is able to collapse at variable rates, but regardless of when collapse occurs, the duration for which the AMOC remains in a collapsed state is the same. In this figure, we move HF0.2_100 forward by 100 years to align the collapse with that of HF0.125_115. Image generated with Matlab 2020b.

In what follows, we present for the first time, a fully-coupled, proxy-compatible model of the YD with proper boundary conditions. We employ the UofT-CCSM4 model^29^ to simulate the entirety of the YD as caused by the injection of proxy-compatible freshwater forcing in the Arctic Ocean. We find that the 1000-year duration of the YD emerges naturally and that a rapid recovery occurs via the mechanism of a polynya opening in the initial sea ice cover of the Irminger Sea Basin (Fig. 1). Crucially, this is the exact same physical mechanism that was recently shown to have been responsible for the transition from stadial to interstadial conditions in Dansgaard–Oeschger events^30^. Most striking is the agreement between our model and the NGRIP-derived temperature (Fig. 1g). Not only is the magnitude of the temperature drop in strong agreement, but the duration is also correct. Additionally, the slow increase in temperature during the YD as well as the rapid increase at the end of the YD are also captured. Finally, the overshoot coincides with the Preboreal Oscillation. This warming would have increased the rate of deglaciation, providing the freshwater necessary for MWP1B, as well as potentially increasing the rate of precipitation in northern Africa, leading to the onset of the greening of the Sahara. It is important to note that MWP1B does not cause a subsequent reduction in AMOC strength because the entry point into the ocean is far from sites of NADW formation and thus is advected to the south^19^.

## Accurate numerical models of Younger Dryas climate

We examine the relationship between AMOC and freshwater forcing under YD orbital and physical conditions using an ensemble of numerical simulations performed with the UofT-CCSM4 model^29^ (see “Methods”). Each simulation employs a distinct freshwater forcing scenario that is characterized by its duration and strength, but the forcing is always applied at the same location, namely over the Beaufort Sea (see Supplementary Fig. S1). Our experimental setup differs from that employed by Manabe and Stouffer^23–25^ in a number of important ways: firstly, our model includes a dynamical sea-ice component coupled to both the ocean and the atmosphere—a critical requirement for properly modelling the YD. Secondly, we apply the freshwater forcing to the correct location^10,11^. And thirdly, we constrain the total volume of freshwater forcing by examining the associated sea level rise.

We investigate two sets of forcing scenarios: “Low and Slow” where a low forcing strength is applied for a very long duration, and “Hard and Fast” where moderately strong to very strong freshwater fluxes are applied for very short durations. These experiments are described in Table 1. Additional supporting simulations are mentioned as needed.

**Table 1:** Younger Dryas numerical simulations performed with the UofT-CCSM4 model. ESL increase is the estimates increase in ESL with the forcing scenario under the YD ocean geometry.

Simulation name	Forcing strength (Sv)	Forcing length (years)	ESL increase (m)	AMOC collapse
Low and slow				
LS0.05	0.05	800	3.74	No
LS0.1	0.1	800	7.47	No
Hard and fast				
HF0.15_50	0.15	50	0.70	No
HF0.15_60	0.15	60	0.84	No
HF0.15_65	0.15	65	0.91	Yes
HF0.15_75	0.15	75	1.05	Yes
HF0.15_100	0.15	100	1.40	Yes
HF0.125_100	0.125	100	1.17	No
HF0.125_110	0.125	110	1.28	No
HF0.125_115	0.125	115	1.34	Yes
HF0.125_125	0.125	125	1.46	Yes
HF0.2_30	0.2	30	0.56	No
HF0.2_35	0.2	35	0.65	No
HF0.2_40	0.2	40	0.75	Yes
HF0.2_45	0.2	45	0.84	Yes
HF0.2_50	0.2	50	0.93	Yes
HF0.2_60	0.2	60	1.12	Yes
HF0.2_100	0.2	100	1.87	Yes
HF0.6_10	0.6	10	0.56	Yes
HF6_1	6	1	0.56	Yes
HF4_1	4	1	0.37	No
HF3_1	3	1	0.28	No
HF2_2	2	2	0.37	No
HF1_5	1	5	0.47	Yes

### Low and Slow

**Figure 2:**
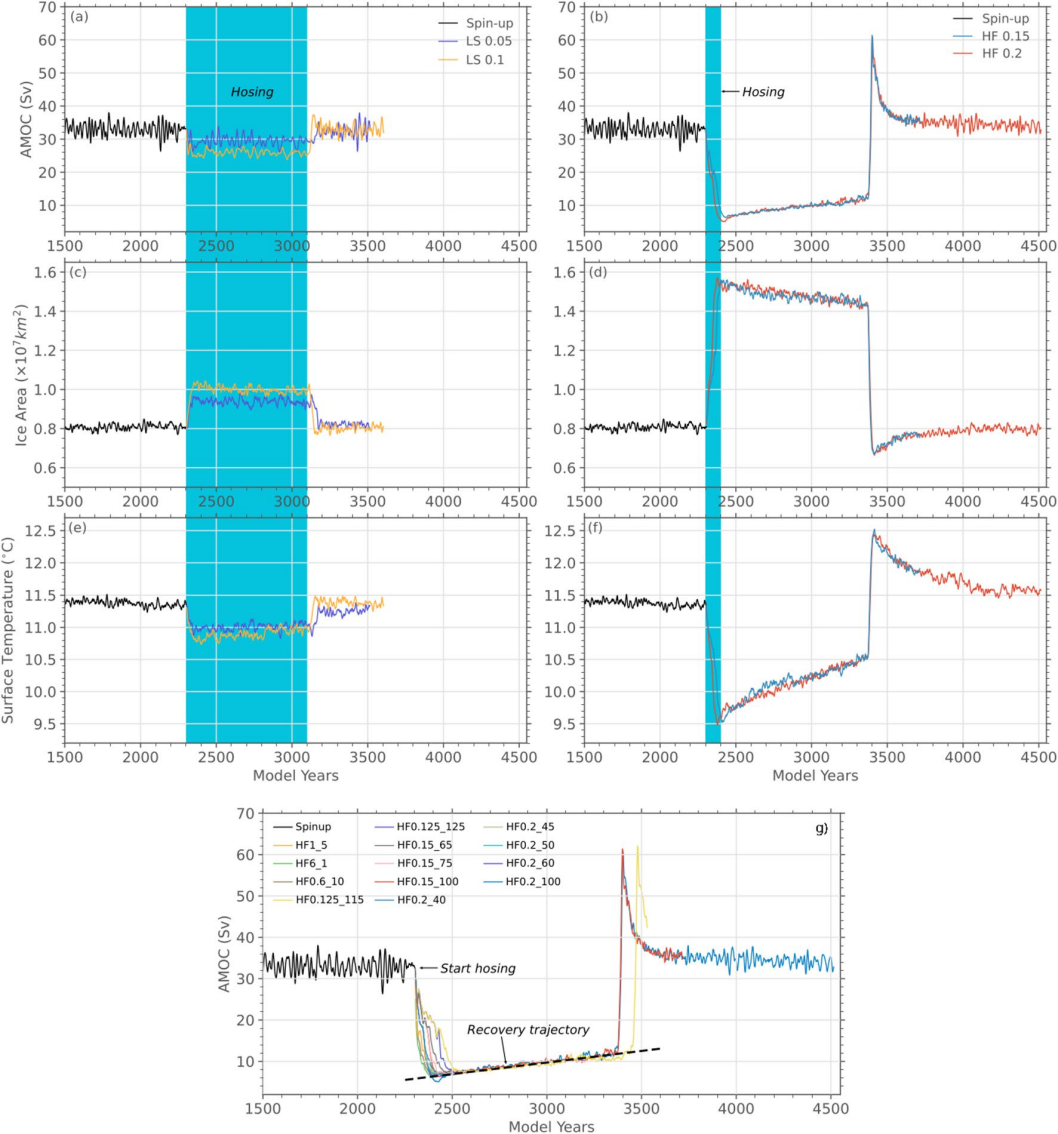
AMOC strengths (**a**,**b**), northern hemisphere sea ice areas (**c**,**d**) and global mean surface temperatures (**e**,**f**) in selected YD models with different forcing scenarios. The left column shows the response of our climate model in the ‘Low and Slow’ forcing scenario to a 0.05 Sv and 0.1 Sv forcing applied for 800 years. The right column shows the response in the ‘Hard and Fast’ scenario when either a 0.15 Sv or 0.2 Sv forcing is applied for 100 years. The forcing intervals are highlighted in blue. Panel (**g**) shows the AMOC response in all our ‘Hard and Fast’ forcing simulations that result in a collapse of the AMOC. In all simulations, the freshwater forcing was applied in the Beaufort Sea with strength and duration as indicated in the simulation name in the legend and in Table 1. After collapsing all simulations seem to follow an identical trajectory towards AMOC recovery that is characterized by the opening of a polynya in the Irminger Sea and a sharp increase in the AMOC strength.

**Figure 3:**
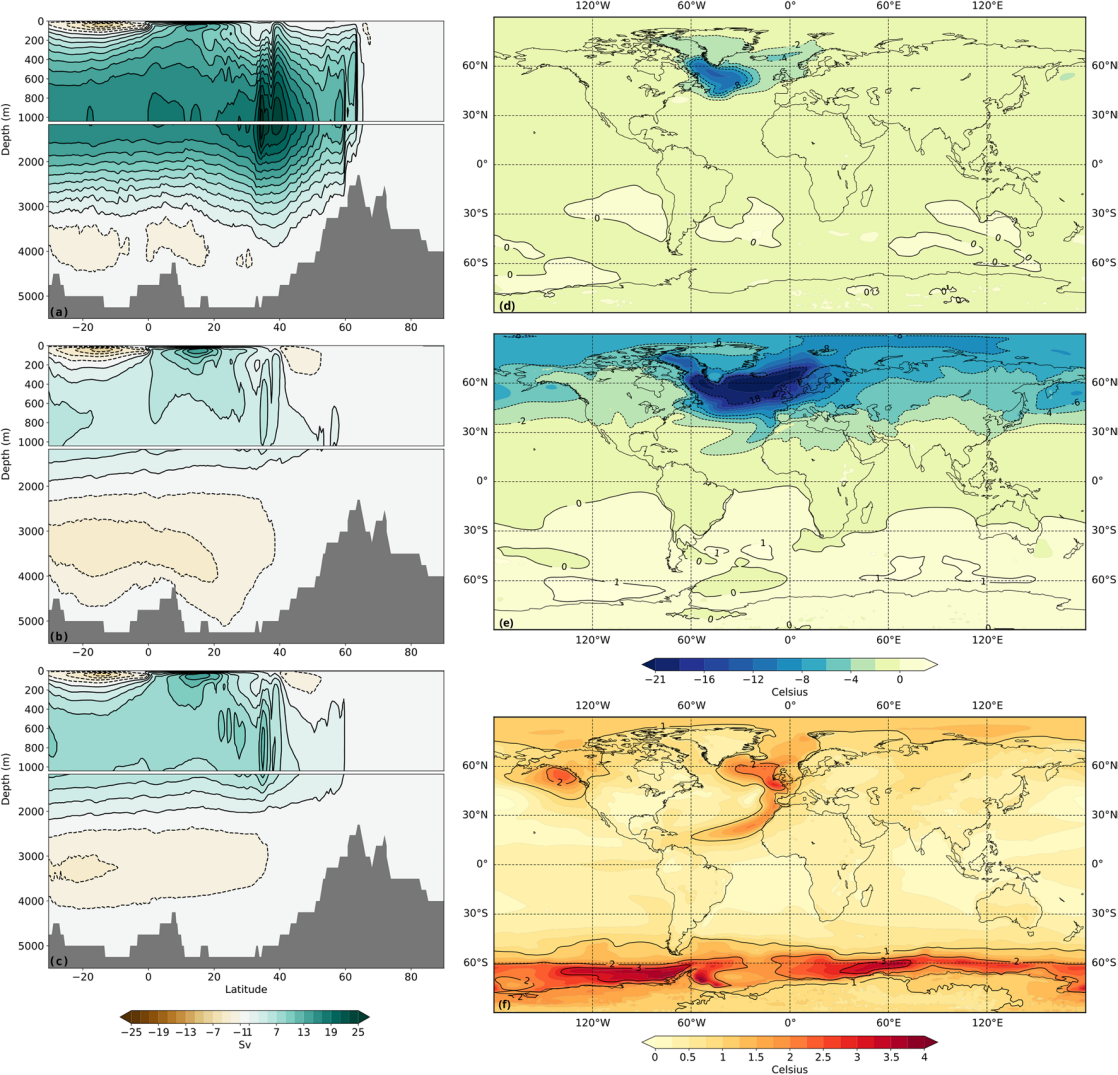
AMOC streamfunctions in (**a**) LS0.1 (years 2501–2700), and in HF0.2_100 (**b**) immediately after the termination of freshwater forcing (years 2401–2600) and (**c**) immediately before the transition from the off-state to the on state (years 3101–3300). In the HF0.2_100, and in other HF simulations that collapse, the NADW formation comes to a halt leading to the disappearance of the top overturning cell. A shallow residual overturning circulation remains that largely arises from the surface wind forcing and the counter-clockwise rotation of a now stronger and expansive bottom cell. Over the  1000-year shut-down of the AMOC, the top cell begins to strengthen. Temperature anomalies with the pre-industrial are also shown for (**d**) LS0.1 (years 2501–2700) and (**e**) HF0.2_100 (years 2401–2600). (**f**) shows the temperature anomaly between years 3101–3300 and 2401–2600 of HF0.2_100. Image generated with Panoply 5.0.5.

The first forcing scenario we consider is called ‘Low and Slow’ in which very low strength freshwater forcing of different magnitudes are applied over the Beaufort Sea gyre for 800 years. This scenario replicates the continuous freshwater forcing configuration that has been employed in existing studies for the YD^23,24,31^. Two simulations, namely LS0.05 and LS0.1, with forcing strengths 0.05 Sv and 0.1 Sv were performed (Table 1). The AMOC did not collapse in either case and continued to exist in the ‘on’ state for the duration of the forcing, and recovered back to spinup level as soon as the forcing was halted (Figs. 2, 3). It is clear that to achieve the millennium scale of the YD in a low-strength scenario, forcing would be required for the entire duration of the YD. This would lead to an ESL rise that would greatly exceed ESL constraints. For instance, even in the LS0.05 simulation with the lowest forcing strength, continuously forcing for the 1200-year YD duration would imply an ESL increase of 5.6 m, which is far larger than the 2–3 m can be inferred from the Barbados Coral Record^3,4^.

The depressed AMOC climatic states, that are obtained in our Low and Slow scenarios, are associated with an 18–20% increase in sea ice coverage in the NH and a $\sim \, 4\%$ reduction in global mean surface temperatures (Fig. 2). Regionally, the cooling is almost entirely contained over the Greenland–Iceland–Norwegian Seas with the western North Atlantic seeing greater cooling than the eastern North Atlantic (Fig. 3). Cooler temperatures are also seen over large parts of Greenland where temperatures drop by $\sim -2\,^\circ$C. This pattern and magnitude of surface cooling are incompatible with the observed widespread cooling over much of North America and Europe^1^. Additionally, the cooling over Greenland is much less than what is determined using the NGRIP $\delta ^{18}O$ proxy record^32^. When forcing is terminated, the NH sea ice area and the global mean temperature rapidly return to pre-forcing levels.

### Hard and Fast

The “Hard and Fast” set includes a large number of simulations where both the forcing strength and the forcing duration were varied over an order of magnitude (Table 1). In this scenario, the application of freshwater forcing resulted in a considerably larger reduction of the AMOC strength compared to the LS scenarios, but in several simulations, the AMOC mounted a recovery as soon as the forcing ceased (Supplementary Fig. S3). However, in several other simulations, the injection of the freshwater forced AMOC to transition into an ‘off’ state from which it could not immediately recover after the termination of the forcing (Fig. 2. Here, we define an ‘off’ state as one where the AMOC top cell involving the formation of the North Atlantic Deep Water has essentially shut down (Fig. 3).

As is seen in the case of HF0.2_100 and HF0.15_100 simulations (Fig. 2), after the collapse the AMOC stays in the off state for almost a millennia before experiencing a rapid transition back to the ‘on’ state. This transition involves the opening of a polynya in the Irminger Sea that reactivates the air–sea exchange of heat flux and momentum and leads to a loss of surface buoyancy, a process similar to that previously described in this model for transition from the stadial to interstadial state during a Dansgaard–Oeschger Oscillation^30^.

As in the case of LS simulations, the global mean temperature and the NH sea ice area evolve in lock-step with changes to AMOC (Fig. 2). However, because of the shut-down of the AMOC, NH temperatures decrease considerably more than in any LS simulation and the cooling is much more widespread (Fig. 3e). During the period over which the AMOC stays shut down, temperatures in the southern hemispheres begin to increase due to the shift in deep water formation to the Southern Ocean (Fig. 3f). Similarly, but to a lesser extent, temperatures in north-eastern sections of the Pacific and the Atlantic also increase during the shut-down as deep water formation begins to slowly resume in the NH.

We note that although the forcing is applied over the Arctic, where the freshwater which caused the YD is known to have entered the ocean, the dynamical evolution of our model does not change if the same forcing is applied over the Ruddiman Belt in the North Atlantic, see Supplementary Fig. S1. This experiment is simply a recreation of previous sensitivity tests^27^ for studying the response of a model to a conventional AMOC forcing region and not based on any expected meltwater pathway. However, the results attest to the fact that the response of the climate system is governed by the physical boundary conditions and is not particularly sensitive to the location of the forcing so long as there is a clear pathway for the meltwater to infiltrate the deep water formation regions. See Supplementary Section A.

A total of 13 simulations from our HF set exhibited collapse in response to freshwater forcing, but for reasons of computational tractability, only three simulations were run out past the full recovery (see Supplementary Materials). Nevertheless, two important observations can be made about the dynamics of this highly non-linear system. Firstly, regardless of the forcing duration or the forcing strength, all collapsed simulations fall onto the same ‘collapse trajectory’. To illustrate this point, consider HF0.2_100 which is one of our faster-collapsing simulations, and HF0.125_125 which is our slowest-collapsing simulation. Even though the latter collapses 100 years after the former, it reaches a minimum AMOC that falls exactly on the same trajectory taken by HF0.2_100. Furthermore, the model continues its subsequent evolution along the same trajectory as HF0.2_100. Secondly, comparing the same two simulations shows that regardless of when the full collapse occurs (i.e. model year), the AMOC stays shut for nearly a millennium before recovering.

## Preferred model of the Younger Dryas

The Barbados coral reef derived record of sea level change constrains the ESL increase to no more than 5 m over the course of the YD. This includes contributions not only from the lake outburst that caused the YD, but also from the constant background melt derived from the ice sheets existing at that time. Consequently, the volume of freshwater released from the lake outburst can be at most 5 m ESL equivalent, and very likely much less than that. The ESL equivalent freshwater input that collapses the AMOC in our 13 YD simulations ranges from 0.47 to 1.87 m, with 7 scenarios being< 1 m. Therefore, simply from volume considerations all our collapsing HF simulations constitute plausible YD models. However, the peak flow rate and the duration over which that rate was maintained is not very well constrained. There is limited evidence from proxy analysis that indicate that the peak flow rate could have been 0.22 Sv^10^. This suggests that the HF models with 0.2 Sv of sustained forcing are most likely and we choose HF0.2_40 as our preferred model as it simulates the YD with the least amount of freshwater input (0.75 m ESL rise) at that forcing strength.

**Figure 4:**
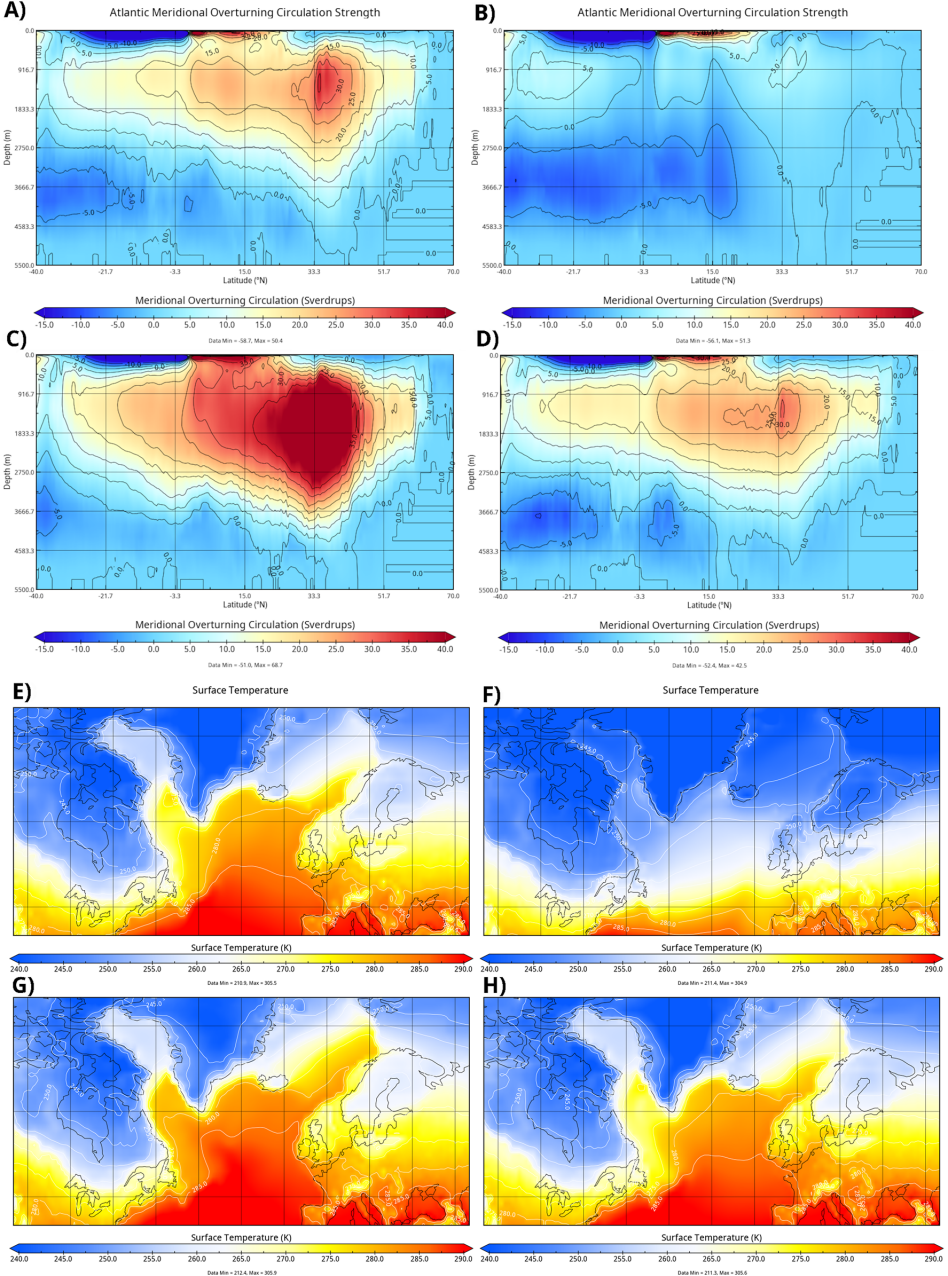
In panels (**A**) through (**D**), the strength of the AMOC is shown before collapse (model year 2301), after collapse (model year 2400), during peak overshoot (model year 3400), and after recovery (model year 3900). In panels (**E**) through (**H**) the surface temperature is shown at the same points as the first part of the figure. Image generated with Panoply 5.0.5.

The HF0.2_40 model was not run through the YD recovery, however, given the observation that the dynamics of all models in which the AMOC collapses follow the same collapse trajectory (Fig. 2g), we can with reasonable confidence study the recovery of HF0.2_100 model instead. The picture of YD that emerges is as follows: the AMOC collapses from the freshwater forcing (Fig. 4A,B), and remains in the off state without any additional forcing for a millennium, precisely the observed duration of the YD. This leads to profound cooling over Northern and Western Europe and over North America (Fig. 4E,F). Recovery from the YD occurs through the opening of a polynya in the Irminger sea during NH winter (Fig. 1) which leads to a dramatic increase in the strength of the AMOC (Fig. 4C) and NH temperatures (Fig. 4G). Subsequently, the strength of the AMOC streamfunction recedes to pre-YD level and the temperatures over the NH cool slightly (Fig. 4D,H). The simulated evolution of temperature at NGRIP over Greenland during the YD is found to match the observed temperature change quite well (Fig. 1).

## Conclusion

The Younger Dryas was a return to glacial conditions during the latter stages of the most recent termination and caused widespread cooling and changes to precipitation patterns across large swaths of North America and Europe at the dawn of the Holocene. Further south, it is also likely that the YD produced a dry period during the most recent African Humid Period (Green Sahara) the recovery from which provided a mechanism to synchronize its expression across Africa. These regional effects had a profound influence on the lives of humans in multiple regions^33–36^. A compelling explanation of the dynamics underpinning the YD is therefore important, not only for understanding the YD itself but also, for understanding the anthropological impacts of YD in the early Holocene.

So far, no adequate explanation of the dynamics of the YD, originating as a result of the injection of freshwater in the oceans, has emerged because modelling studies have either not applied the model boundary conditions with acceptable accuracy, or used simplified and coarse climate models. A much larger issue with existing studies has been that they apply continuous freshwater forcing for the duration of the YD in order to keep the AMOC depressed and thereby maintain an artificially induced climate system. The forcing scenarios employed in these studies are unrealistic because they greatly exceed the ESL constraints for the total rise in sea level that could have occurred during the YD. Therefore, it has always remained an unresolved question whether or not the YD was maintained for the entire duration by continuous freshwater induced suppression of the AMOC.

Here we have shown that it is possible to recreate the millennium time-scale of the YD without violating ESL constraints or using continuous freshwater forcing for the entire duration. Furthermore, we show that the AMOC collapse is dependent on the total volume of freshwater forcing, with smaller total volumes required when very intense forcings are applied over short periods of time (see Supplementary Fig. S4). This leads to a set of plausible models with different total freshwater volumes that can be narrowed further if future studies are able to provide tighter bounds on the duration and peak strength of the lake outburst event.

The recovery from YD is found to involve the opening of a polynya in the Irminger Sea, via a mechanism that is identical to that which has been proposed for the transition from the stadial to interstadial state during the Dansgaard–Oeschger oscillations^30^. This mechanism provides an extremely rapidly which is in line with evidence from ice cores^37^. This suggests a possible link between the YD and Dansgaard–Oeschger oscillations that would be explored further in a future study. The increase and overshoot in the strength of the AMOC during recovery is likely to create conditions for the Pre-Boreal Oscillation^6,7^. Additionally, the overshoot in NH temperatures^38^ may have led to increased meltwater production, providing a source for MWP1B. Finally, we show that the AMOC is very sensitive, collapsing or recovering with only a small change in either forcing duration or forcing strength.

The combination of the constraints provided by eustatic sea level rise and AMOC strength demonstrates that the only viable explanation of the YD is that it was caused by an instability of the freshwater run-off pathway during which freshwater was delivered to the Arctic Ocean at a very high rate for a very brief period of time. Our analysis has also demonstrated the critical role in the climate response to this forcing that is played by sea ice dynamics in response to ocean forcing.

Similar to the YD, the 8.2 ka event^39^ also involved an outburst of the proglacial lakes that had formed along the margins of the Laurentide ice sheet^39^. Therefore, to assess the robustness and uniqueness of our results for the YD, we replicated selected forcing scenarios that led to the full collapse of the YD AMOC, under boundary conditions applicable for 8.2 ka. Under all three tested scenarios (Supplementary Fig. S2) the AMOC rebounded as soon as the forcing was eliminated. This is in agreement with proxy reconstructions showing no sustained collapse of the AMOC. As the response of our model is not sensitive to the location of the freshwater forcing, the difference between the responses under YD and 8.2 ka conditions is driven by the differences in topographical and orbital configurations between these events. This finding offers an explanation for why two large proglacial lake outbursts during the recent deglaciation led to two different responses and highlights the important role played by the earth system’s boundary conditions in determining the emergence of non-linear phenomena.

## Methods

In this study we use the UofT-CCSM4 model which is derived from the CCSM4 model^40^ with some modifications^29,41^. In particular, UofT-CCSM4 differs from CCSM4 in the choice of a depth-dependent hyperbolic tangent form for the diapycnal diffusivity, and tidal mixing is left unparametrized. UofT-CCSM4 has been used extensively in the context of paleoclimate modelling for the study of the mid-Pliocene^41–45^, the most recent glacial cycle^29,30,46,47^ and the mid-Holocene^48–50^. The boundary conditions for our YD experiments have been implemented as accurately as possible in our numerical model. The orbital parameters, trace gas concentrations, orography (including ice sheet orography), and bathymetry appropriate for 12.5 ka. The bathymetry, as well as the thickness and spatial distribution of terrestrial ice, is obtained from the ICE-7G (VM7) model of glacial isostatic adjustment^51,52^. However, in order to avoid the challenges that can arise whenever the ocean grid is changed (i.e., the addition or removal of ocean grid cells) we chose to avoid modifying the land-sea mask and instead kept it same as that used in our model for the PMIP4 Last Glacial Maximum (LGM) experiments^47^. The LGM land-sea distribution is nearly identical to that at the YD (this is especially so at the resolution of our global coupled model) except for the fact that the Barents Sea and Kara Sea ice sheets had deglaciated by the time of the YD, leading to the existence of open water over that region. Since we retain the LGM land feature over these regions, in recognition of the fact that there were no ice sheets, we set the elevation over this region to a very small value, so as to minimize the effect on the model.

All simulations in our two forcing sets were started at year 2300 of a baseline model of the YD which was started from present day ocean temperature and salinity conditions and integrated to statistical equilibrium.

### Supplementary Information

Supplementary Information.

## Data Availability

To-do The UofT-CCSM4’s code base is shared with the NCAR CCSM4 model. The latter can be found at https://www.cesm.ucar.edu/models/ccsm/download. Runtime configurations that distinguish the UofT-CCSM4 model from the NCAR CCSM4 model are discussed in “Methods” section. The ICE-7G ice loading histories used for the boundary conditions can be obtained from the website of WRP: https://www.atmosp.physics.utoronto.ca/~peltier/data.php.
