# Supplementary material for: Into the Holocene, anatomy of the Younger Dryas cold reversal and preboreal oscillation
Source: Sci Rep. 2024 Feb 7;14:3134. doi: 10.1038/s41598-024-53591-2 (PMC11291662; doi:10.1038/s41598-024-53591-2)
Supplement: Supplementary file 1 — Supplementary Information. [file 41598_2024_53591_MOESM1_ESM.pdf]

Supplementary Information for: Into the Holocene, Anatomy of the Younger Dryas Cold Reversal and Preboreal Oscillation

Jesse Velay-Vitow[1,*,+], **Deepak Chandan**[1,+], **and W. Richard Peltier**[1]

[1]Department of Physics, University of Toronto, Toronto, ONT, Canada
[*]jvitow@physics.utoronto.ca
[+]These authors contributed equally to this work

ABSTRACT

History of investigation into the YD

Over the last three decades, a consensus view has developed that a release of glacial meltwater from proglacial lakes along the southern and western flanks of the Laurentide Ice Sheet (LIS) formed a freshwater cap over the North Atlantic[1]. The resulting gain in the buoyancy of North Atlantic surface waters sharply reduced the rate of North Atlantic Deep Water (NADW) formation and thus caused a reduction in the strength of the Atlantic Meridional Overturning Circulation (AMOC)[2]. The strength of the AMOC during the YD has been shown to be markedly reduced based on the Pa/Th record[3], nearly as much so as during the prior Heinrich Stadial 1[4] during which it has been argued the AMOC collapsed completely. This strongly affected the inter-hemispheric heat transport and led to a marked reduction in Northern Hemispheric temperatures as well as a dramatic expansion of sea ice[5,6]. It was initially suggested that freshwater had entered into the North Atlantic through the St. Lawrence River outlet, a view advanced by[1] and favoured by Broecker[2]. This was challenged by[7] in which it was argued that there was no evidence of increased meltwater outflow through the St. Lawrence River during the YD.

This led to the suggestion, based upon an analysis of deglacial meltwater runoff from the North American continent, that the meltwater runoff at YD onset had entered the Arctic Ocean through the modern Mackenzie River[8,9], and the effect of the introduction of freshwater into the Arctic Ocean was a significant reduction in the strength of the AMOC[5,6]. The temporal correlation between the sedimentological record, which demonstrated an increased rate of glacial runoff through the Mackenzie River outlet[10] and the sharp reduction in Northern Hemisphere temperature inferred from the Greenland ice core record[11] served as the confirmation that the Northern route was correct. This was further attested by explicit modelling of the boundary currents in the Arctic and North Atlantic[12,13] in which it was shown that any freshwater which entered into the North Atlantic through the St. Lawrence River would be transported to the south, far away from the sites of NADW formation, and thus have no effect on the AMOC. A final piece of evidence was the discovery of a strong $\delta^{18}O$ spike at the onset of the YD in sediment cores raised from the Beaufort Gyre[14,15].

There are a number of inadequacies in these prior attempts, some attributable to technological limitations, and others to incorrect assumptions. First, forcing has often been applied in an incorrect location, usually directly over NADW production sites in the North Atlantic, to directly impact the AMOC. This, however, appears to be insignificant, as Peltier et al.[5] showed that changing the location of forcing from the Atlantic to the Arctic resulted in a minimal effect, specifically a longer lag between initiation of forcing and AMOC slowdown. Second, the amount of freshwater used to simulate the YD has been significantly more than is reasonable based on sea level constraints. The Barbados record[16,17] suggests that ESL rose by a maximum of ~ 5 m during the YD. This is clearly an upper bound on the estimate of the volume of glacial freshwater flowing into the Arctic since this ESL increase also includes all other meltwater contributions from the Laurentide, Fennoscandian, Greenland and Antarctic ice sheets. In the Manabe and Stouffer papers, inferred ESL rise varies between 0.87m to more than 40 m. Third, the conditions of the climate model at YD onset were not accurately specified to those which are known to have existed. This third point includes orbital parameters, trace gas concentrations, orography and ocean bathymetry, and the Land-Sea mask employed. Typically, the climate employed has been similar to last glacial maximum or modern-day For example, Manabe and Stouffer used modern-day climate, with the exception of closing the Bering Strait. Additionally, the terrestrial ice used in the Manabe and Stouffer models has inaccuracies typical of ice models at the time and is of a low resolution. The final shortcoming is that many early attempts have not used fully coupled climate models. As the YD involves land ice, atmosphere, sea ice, and oceans,

any model which ignores a subset of these is insufficient. One final issue with the existing modelling attempts is that while the mechanism of entrance into the YD is now well understood, the extremely rapid exit remains mysterious. The Greenland ice cores provide a record of rapid northern Hemisphere warming, which has been dated to the YD end[18], and which may have occurred over less than two decades[19]. As the end of the YD is temporally correlated with the greening of the Sahara[20] and the Preboreal Oscillation[21,22], a better understanding of the physical mechanism responsible for the recovery from the YD would provide crucial insight into the climatological conditions at the beginning of the Holocene.

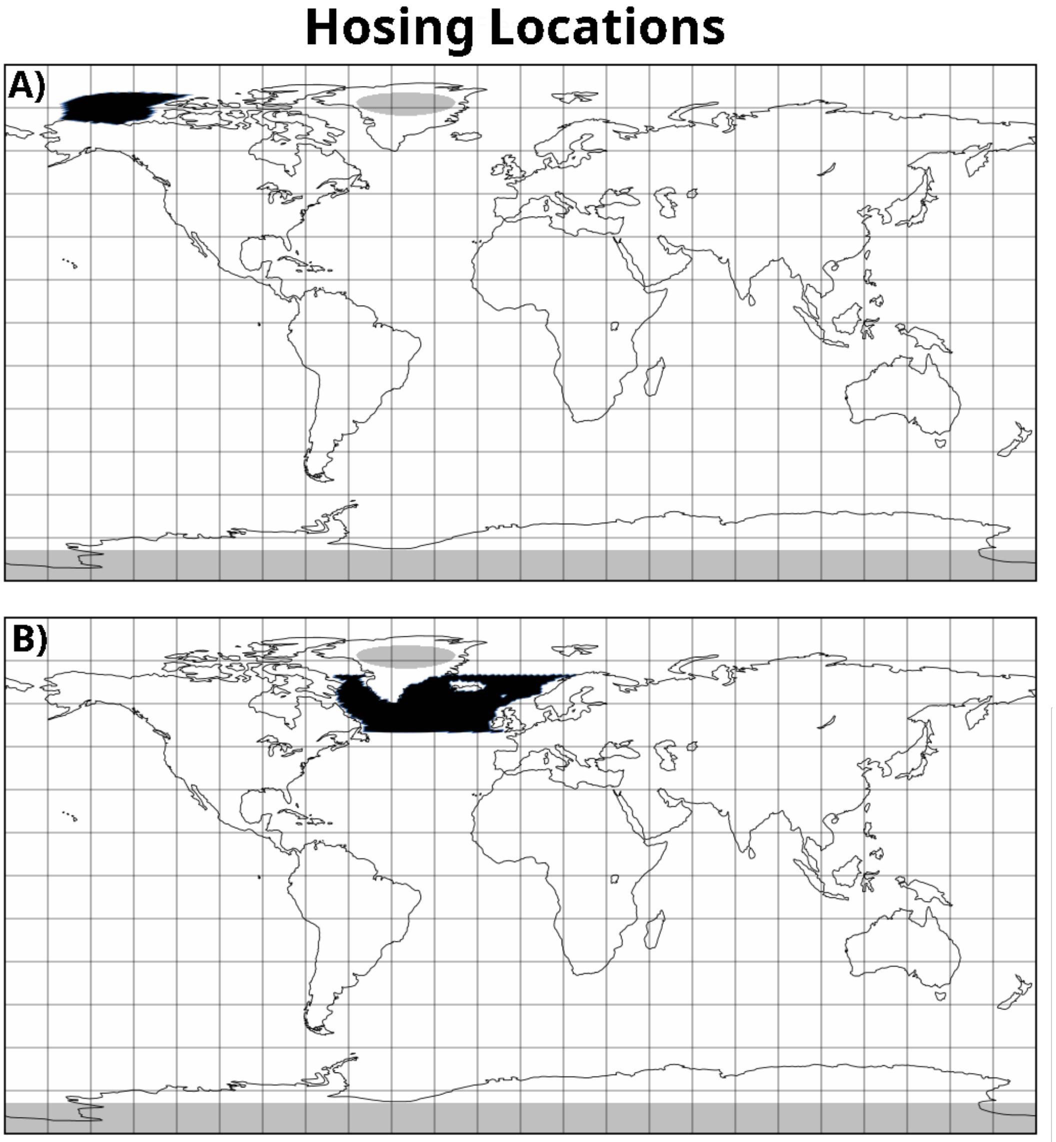

Figure S1. In panel A) we see the region which is hosed in the Arctic forcing scenarios, the Beaufort Sea. In panel B) the hosing region for the Atlantic scenarios, the Ruddiman Belt, is shown.

Rational for conducted tests

The decision to separate tests into Hard and Fast (HF) and Low and Slow (LS) was based on the observation that nearly all attempts to model the YD involved persistent forcing for the entire duration, while all sedimentological[10,14] and proxy evidence[8,9] points towards a rapid outburst. In designing the tests, we chose three initial forcing levels, 0.05 Sv, 0.1 Sv and 0.2 Sv. Upon realization that the weaker two did not collapse even after a millennium of forcing, but that the stronger collapsed after a century, we began to run a suite of sensitivity tests on this collapse. This led to bifurcating the forcing twice, to 0.15 sv

and then 0.125 sv, and then reducing forcing by 5 years at a time until the shortest duration which caused a collapse was found. See below for a full discussion of the sensitivity profile. The additional experiments with even stronger forcing were conducted to fully determine the high forcing, low duration portion of the sensitivity phase space. These values were chosen to allow direct comparison with the 8.2 ka event, discussed subsequently.

Comparison with the 8.2 ka event

Similar to the YD, the 8.2 ka event also involved an outburst of the proglacial lakes that had formed along the margins of the Laurentide ice sheet[23]. The volume of freshwater added to the oceans is expected to have been similar to that during the YD. However, unlike the YD, this event did not lead to a prolonged shutdown of the AMOC. Rather the AMOC strength and the surrounding temperatures decreased rapidly during the injection of freshwater and then rebounded quickly following the termination of the forcing. While in the case of the 8.2 ka event, the freshwater was released through the Hudson Strait into the Labrador Sea rather than into the Arctic, a more crucial difference between the two relates to the surface boundary conditions; the Northern Hemisphere ice sheet configuration was significantly different at 8.2 ka than during the YD and the Bering Strait and the Canadian Archipelago were open. We applied three HF forcing scenarios that caused a collapse of the YD AMOC to 8.2ka conditions. In all cases the AMOC started returning to pre-forcing levels as soon as the forcing was ceased (Fig. S2).

Phase space of AMOC collapse

The large and comprehensive set of forcing experiments that we have completed allow us to examine the phase space between AMOC on and off states, and help us further clarify the conditions under which AMOC collapse can be observed (Fig. S4). As no collapse occurred in certain cases, even for extremely long durations of forcing, it is evident that there is a forbidden region in the phase diagram. We've determined this to be around 0.11 Sv, as 0.125 Sv collapses in 115 years, and 0.1 Sv fails to collapse even after 1000 years of forcing. From this, we can deduce that there is a maximum time to collapse, which is most likely 125 years. We can also bound the phase diagram from above by physical constraints on flow rates derived from estimates of lake volume. In our models, the strongest forcing we used was 6 Sv for a single year, which is an upper bound on forcing strength. For simplicity's sake, we take a single year as our minimum forcing duration. These four constraints fully bound the allowed phase space.

References

1. Rooth, C. Hydrology and ocean circulation. *Prog. Oceanogr.* **11**, 131–149 (1982).
2. Broecker, W. S. *et al.* Routing of meltwater from the Laurentide Ice Sheet during the Younger Dryas cold episode. *Nature* **341**, 318 (1989).
3. McManus, J. F., Francois, R., Gherardi, J.-M., Keigwin, L. D. & Brown-Leger, S. Collapse and rapid resumption of Atlantic meridional circulation linked to deglacial climate changes. *nature* **428**, 834–837 (2004).
4. Süfke, F. *et al.* Constraints on the northwestern Atlantic deep water circulation from 231Pa/230Th during the last 30,000 years. *Paleoceanogr. Paleoclimatology* **34**, 1945–1958 (2019).
5. Peltier, W., Vettoretti, G. & Stastna, M. Atlantic meridional overturning and climate response to arctic ocean freshening. *Geophys. Res. Lett.* **33** (2006).
6. Peltier, W. Rapid climate change and arctic ocean freshening. *Geology* **35**, 1147–1148 (2007).
7. de Vernal, A., Hillaire-Marcel, C. & Bilodeau, G. Reduced meltwater outflow from the Laurentide ice margin during the Younger Dryas. *Nature* **381**, 774–777 (1996).
8. Tarasov, L. & Peltier, W. R. Arctic freshwater forcing of the Younger Dryas cold reversal. *Nature* **435**, 662 (2005).
9. Tarasov, L. & Peltier, W. R. A calibrated deglacial drainage chronology for the North American continent: evidence of an Arctic trigger for the Younger Dryas. *Quat. Sci. Rev.* **25**, 659–688 (2006).
10. Murton, J. B., Bateman, M. D., Dallimore, S. R., Teller, J. T. & Yang, Z. Identification of Younger Dryas outburst flood path from Lake Agassiz to the Arctic Ocean. *Nature* **464**, 740 (2010).
11. Rasmussen, S. O. *et al.* A new greenland ice core chronology for the last glacial termination. *J. Geophys. Res. Atmospheres* **111** (2006).
12. Condron, A. & Winsor, P. Meltwater routing and the Younger Dryas. *Proc. Natl. Acad. Sci.* **109**, 19928–19933 (2012).
13. Hill, J. C. & Condron, A. Subtropical iceberg scours and meltwater routing in the deglacial western North Atlantic. *Nat. Geosci.* **7**, 806–810 (2014).

14. Keigwin, L. *et al.* Deglacial floods in the beaufort sea preceded younger dryas cooling. *Nat. Geosci.* **11**, 599–604 (2018).

15. Velay-Vitow, J. & Richard Peltier, W. Out of the ice age: Megatides of the arctic ocean and the bølling-ållerød, Younger Dryas transition. *Geophys. Res. Lett.* **47**, e2020GL089870 (2020).

16. Fairbanks, R. G. A 17,000-year glacio-eustatic sea level record: influence of glacial melting rates on the younger dryas event and deep-ocean circulation. *Nature* **342**, 637–642 (1989).

17. Peltier, W. & Fairbanks, R. G. Global glacial ice volume and last glacial maximum duration from an extended barbados sea level record. *Quat. Sci. Rev.* **25**, 3322–3337 (2006).

18. Severinghaus, J. P., Sowers, T., Brook, E. J., Alley, R. B. & Bender, M. L. Timing of abrupt climate change at the end of the younger dryas interval from thermally fractionated gases in polar ice. *Nature* **391**, 141–146 (1998).

19. Alley, R. B. *et al.* Abrupt increase in greenland snow accumulation at the end of the younger dryas event. *Nature* **362**, 527–529 (1993).

20. Demenocal, P. *et al.* Abrupt onset and termination of the African Humid Period:: rapid climate responses to gradual insolation forcing. *Quat. science reviews* **19**, 347–361 (2000).

21. Björck, S., Rundgren, M., Ingolfsson, O. & Funder, S. The preboreal oscillation around the nordic seas: terrestrial and lacustrine responses. *J. Quat. Sci. Publ. for Quat. Res. Assoc.* **12**, 455–465 (1997).

22. Fisher, T. G., Smith, D. G. & Andrews, J. T. Preboreal oscillation caused by a glacial lake agassiz flood. *Quat. Sci. Rev.* **21**, 873–878 (2002).

23. Barber, D. C. *et al.* Forcing of the cold event of 8,200 years ago by catastrophic drainage of Laurentide lakes. *Nature* **400**, 344–348 (1999).

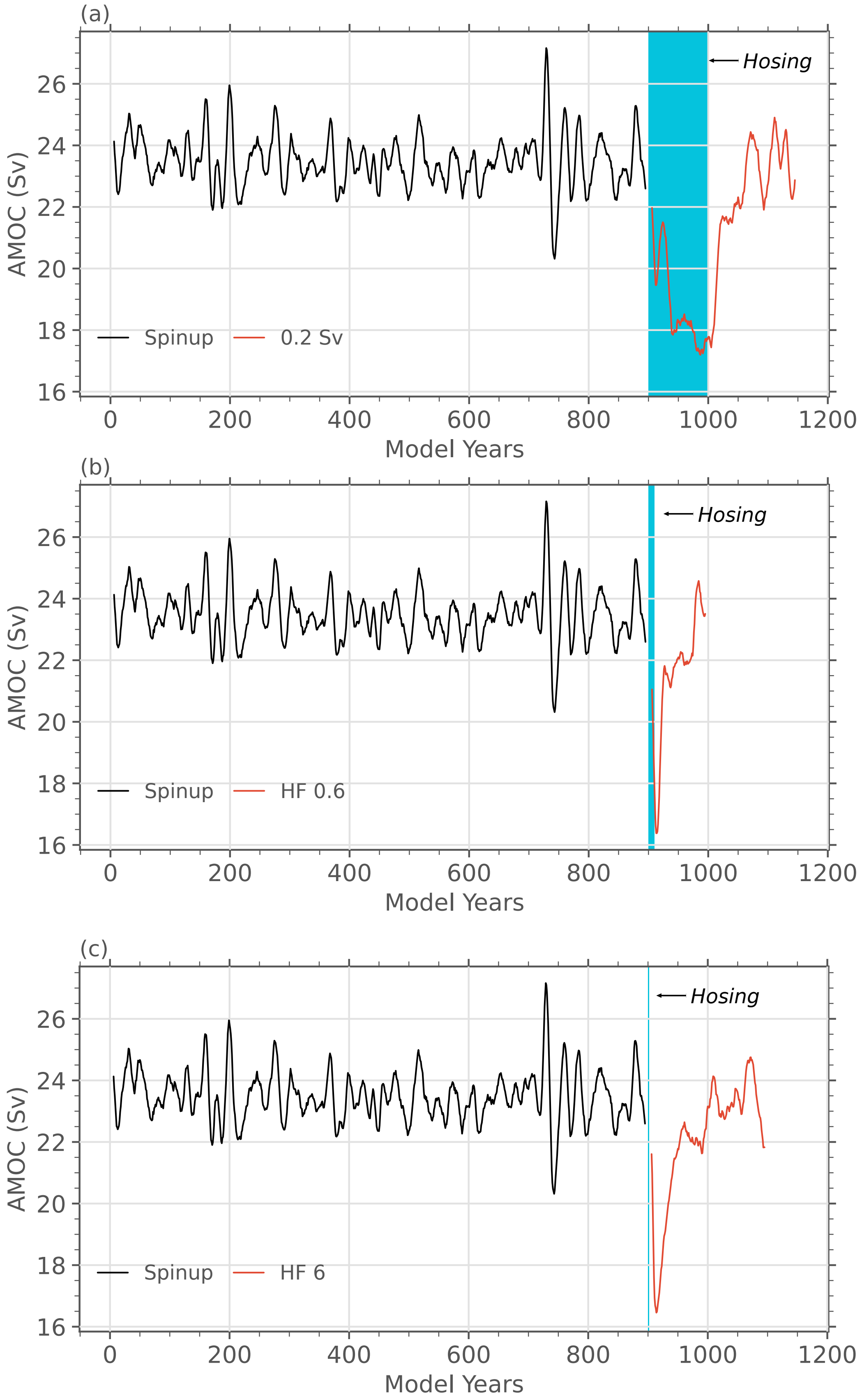

Figure S2. Panel (a), (b) and (c) show the impact of 0.2 sv, 0.6 sv and 6 sv of fresh water forcing applied to the model over the Arctic ocean using correct boundary conditions for 8.2 ka. As no sustained collapse occurs, we can conclude that the specific boundary conditions of the YD are required to observe a prolonged AMOC collapse.

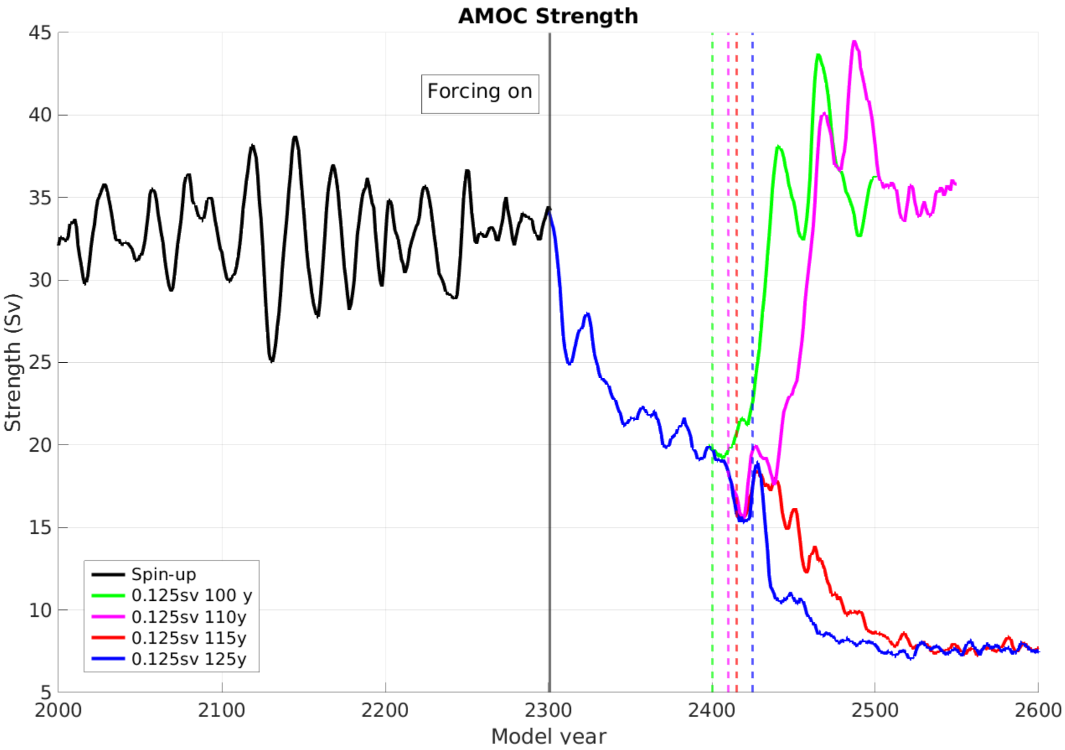

Figure S3. Here we see several forcing scenarios which recover immediately after forcing is ceased, and slightly longer duration forcing scenarios under which collapse occurs.

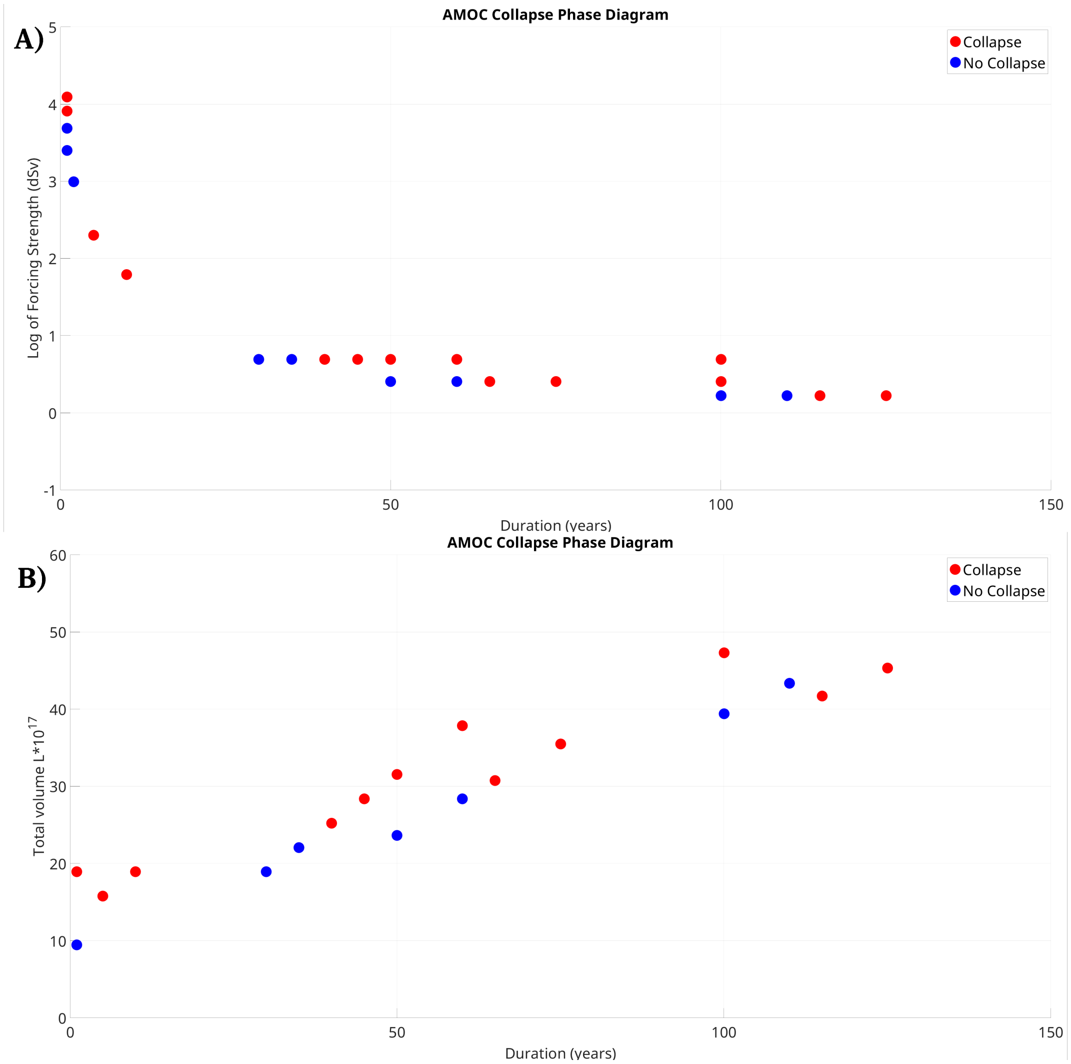

Figure S4. Phase diagrams for the response of the AMOC in our set of forcing runs. The phase space formed by the forcing strength and the forcing duration demonstrates a strongly non-linear boundary between the region where AMOC collapses and the regions where it does not (a). When the responses in our simulations are plotted in the phase space formed by the total volume of freshwater and the forcing duration then a linear phase boundary is observed (b). The stronger the forcing, the less total volume of freshwater is required to cause a collapse.
